# Sex-specific contemporary trends in incidence, prevalence and survival of patients with non-valvular atrial fibrillation: A long-term real-world data analysis

**DOI:** 10.1371/journal.pone.0247097

**Published:** 2021-02-18

**Authors:** Arthur Shiyovich, Gabriel Chodick, Liat Azani, Matanya Tirosh, Mony Shuvy, David Pereg, Amos Katz, Sa’ar Minha

**Affiliations:** 1 Rabin Medical Center, Petah Tikva, Israel; 2 Sackler School of Medicine, Tel Aviv University, Tel Aviv, Israel; 3 Kahn-Sagol-Maccabi Research and Innovation Institute, Maccabi Healthcare Services, Tel Aviv, Israel; 4 Medical Affairs Pfizer Israel, Herzliya, Israel; 5 Heart Institute, Hadassah Hebrew University Medical Center, Jerusalem, Israel; 6 Department of Cardiology, Meir Medical Center, Kfar-Saba, Israel; 7 Faculty of Health Sciences, Ben-Gurion University of the Negev, Beer-Sheva, Israel; 8 Department of Cardiology, Shamir Medical Center, Zerifin, Israel; King’s College London, UNITED KINGDOM

## Abstract

**Introduction:**

Atrial fibrillation (AF) is a major increasing public health problem worldwide, with clinical and epidemiological differences between men and women. However, contemporary population-level data on incidence and survival are scarce.

**Aim:**

To evaluate sex-specific contemporary trends in the incidence, prevalence, and long-term survival of non-valvular AF in a real-world setting

**Methods:**

AF patients diagnosed between 2007–2015, insured by a large, state-mandated health organization in Israel (Maccabi Healthcare Services) were included. AF was diagnosed based on registered diagnoses. Patients with valvular disease, active malignancy, cardiac surgery ≤ 6 months, or recent pregnancy, were excluded. Annual incidence rate, period prevalence, and 5-year survival for each calendar year during the study period, were calculated.

**Results:**

A total of 15,409 eligible patients (8,288 males, 7,121 females) were identified. Males were more likely to be younger, have higher rates of underlying diseases (ischemic heart disease, heart failure, and chronic obstructive pulmonary disease), but with lower rates of hypertension and chronic kidney diseases as compared to female patients. During the study period, age-adjusted incidence decreased both in men: (-0.020/1,000-person year, p-for trend = 0.033) and, women (-0.025/1,000 person-year p = 0.009). The five-year survival rate was significantly higher among men vs. women (77.1% vs. 71.5%, respectively, p<0.001). Age-adjusted prevalence increased significantly among men (+0.102 per year, p-for trend<0.001) yet decreased among women (-0.082 per year, p-for trend = 0.005). A significant trend toward improved long-term survival was observed in women and not in men.

**Conclusions:**

The current study shows significant sex-related disparities in the incidence, prevalence, and survival of AF patients between 2007–2015; while the adjusted incidence of both has decreased over-time, prevalence and mortality decreased significantly only in women.

## Introduction

Atrial fibrillation (AF) is the most frequent cardiac arrhythmia of clinical significance, with significantly greater prevalence in older persons and those with cardiovascular risk factors and co-morbidities [1, 2]. AF is considered a major public health problem associated with increased risk of thromboembolism and stroke [1, 3, 4], chronic kidney disease, dementia, acute myocardial infarction, heart failure, and mortality [5–9]. It has been estimated that AF accounts for an annual expenditure of $6–26 billion in the United States [10]. The most significant predictors of AF are age and sex; the prevalence of AF doubles with every decade and is 1-2-fold greater in men compared with women [11–13]. Recent reports from different countries demonstrated a trend of increase in the incidence and prevalence of AF over time while mortality seems to be decreasing [5, 7, 14–19]. Furthermore, it has been suggested that these temporal changes are disproportionate between the sexes and different age groups [5, 19, 20]. However, these reports are based mostly on registries and cohort studies and thus might be biased while data from unselected populations are sparse.

The aim of this study was to evaluate sex-specific contemporary trends in the incidence, prevalence and long-term survival of AF patients in a real-world setting.

## Methods

### Study design and population

The current study included patients insured by Maccabi Healthcare Services (MHS), one of the largest healthcare providers in Israel, covering over 2 million members, diagnosed with AF between 2007 and 2015, and aged 21 years or older at the time of diagnosis. The date of AF diagnosis was defined as the index date.

A subject was considered to have AF if they had a registered diagnosis (International Classification of Diseases, Ninth Revision (ICD-9) 427.3x –atrial fibrillation and/or atrial flutter, which will be referred henceforth as AF) documented in one of the following: a hospital discharge letter, cardiologist visit, primary care physician visit (recorded on two separate visits or more), or single documentation by any physician combined with the prescription of oral anticoagulants (OACs). Exclusion criteria were—1. valvular AF—history of heart valve replacement (ICD 9 codes: V42.2, V43.3, 35.2x) and rheumatic mitral stenosis (394.0, 394.1); (hence AF in this manuscript refers to non-valvular AF); 2. active malignancy; 3. cardiac surgery or acute pulmonary heart disease (415.x) in the 6 months before diagnosis; 4. pregnancy within 16 months before AF diagnosis.

The study protocol was approved by the Maccabi Healthcare Services Institutional Review board (0046-16-BBL) and conducted in accordance with the Helsinki declaration. Patient consent was waived by the review board.

### Data collection and definitions

Demographic and clinical data were collected from MHS and Israel’s ministry of health databases. Baseline comorbidities and risk score calculations were obtained from MHS patient registries, described previously [21, 22] and included: age, sex, socioeconomic status (SES), smoking status, hypertension, diabetes mellitus, peripheral vascular disease (PVD), ischemic heart disease (IHD) with or without a history of myocardial infarction (MI), congestive heart failure (CHF), chronic obstructive pulmonary disease (COPD), previous stroke or transient ischemic attack (TIA). The CHA_2_DS_2_-VASc score was calculated for all patients by assigning 2 points for age≥75 years (A2) and history of stroke, TIA, or thromboembolism (S2) and 1 point for each of the following criteria: CHF (C), hypertension (H), diabetes mellitus (D), age 65 to 75 years (A), vascular disease (VA) (defined as previous myocardial infarction, complex aortic plaque, carotid stenosis, and peripheral artery disease) and female sex category (Sc). Data regarding medical therapy (at least 2 dispensed packs during 120 days before index date) were obtained and classified according to the following therapeutic agents: angiotensin-converting enzyme inhibitors (ACE-i), angiotensin receptor blockers (ARBs), alpha-blockers, beta-blockers, calcium channel blockers, digoxin, diuretics, heparins, nitrates, antiplatelets, and vasodilators.

Dates of death or leaving the health maintenance organization (HMO) are reported to MHS daily from the Israel National Insurance Institute. Residential area SES level was based on a commercial index (developed by Points Location Intelligence) ranging from 1 (lowest) to 10 (highest). Scoring is conducted according to various socioeconomic indices (e.g. credit card use information, housing prices, education level, etc.) and was previously shown to highly correlate with the SES index provided by Israel’s National Bureau of Statistics [23].

### Statistical analysis

Parametric and non-parametric variables are summarized as mean ± standard deviation (SD) and median [interquartile range (IQR)], respectively. For categorical values, counts (%) were calculated. Comparisons between sexes were conducted using the Mann-Whitney test or Chi-square test for continuous and categorical variables, respectively. Prevalent AF cases were defined as MHS members alive at the beginning of an index year and before or throughout that year. Incident cases were defined as MHS members diagnosed with AF during an index year. Kaplan-Meier analysis was performed to study the differences in all-cause mortality survival from the index date. We employed a log-linear regression model to estimate annual percentage change, in which age and calendar year included as independent variables. Survival analysis was performed with the use of Cox’s regression models. For the multivariable Cox proportional hazards regression, results are presented as hazard ratios (HR) with 95% confidence intervals (CIs). Two-tailed significance was defined as p<0.05. For survival analysis, patients were classified into 3 groups according to the diagnosis period; 2007–2009, 2010–2012, and 2013–2015. Statistical Analyses were performed with IBM SPSS for windows version 25.0 Armonk, NY: IBM Corp. and R3.5.1.

## Results

### AF incidence

Throughout the follow-up period, a total of 15,409 subjects (8,288 males, 7,121 females) were diagnosed with AF. S1 Table presents the number of incident AF cases by year and sex.

Table 1 displays the baseline characteristics of the incident cases stratified by sex. Male patients (53.8% of all patients) were significantly younger and from higher SES. Furthermore, male patients had increased rates of most cardiovascular risk factors and lower CHA_2_DS_2_-VASC score (2.4±1.9 vs. 3.8±1.7, p<0.001, respectively). The prevalence of chronic medical therapy with antiplatelet medications was significantly higher among males; however; treatment with angiotensin-receptor blockers, beta-blockers, diuretics, and calcium channel blockers was statistically significantly less common compared with females.

**Table 1:** Baseline characteristics of incident cases of AF.

Baseline characteristics	Total	Males	Females	p-value
	(N = 15,409)	(n = 8288)	(n = 7121)	Males vs Females
	% (n)	% (n)	% (n)	
Age (years), Mean±SD		66.8±14.1	72.72±12.94	<0.001
SES*, Median [IQR]	6 [5–7]	6 (5–7)	6 [5–7]	<0.001
Ever smoked*	12.0% (1850)	16.6% (1373)	6.7% (477)	<0.001
CHF	9.3% (1433)	10.4% (859)	8.1% (574)	<0.001
IHD	25.0% (3847)	31.7% (2628)	17.1% (1219)	<0.001
MI	12.2% (1873)	16.6% (1378)	7.0% (495)	<0.001
IHD non-MI	15.0% (2318)	17.9% (1487)	11.7% (831)	<0.001
Stroke	8.2% (1261)	8.1% (675)	8.2% (586)	0.871
TIA	3.9% (606)	3.9% (326)	3.9% (280)	0.999
PVD	5.4% (825)	6.9% (571)	3.6% (254)	<0.001
CKD	27.4% (4228)	25.4% (2109)	29.8% (2119)	<0.001
Diabetes	28.7% (4422)	28.9% (2398)	28.4% (2024)	0.496
Hypertension	70.9% (10927)	65.2% (5403)	77.6% (5524)	<0.001
COPD	2.0% (306)	2.4% (198)	1.5% (108)	<0.001
CHADS_2_ score	2 [1–2]	1 [1–2]	2 [1–3]	<0.001
Median [IQR]				
Medications				
ACE inhibitors	22.3% (3437)	22.2% (1844)	22.4% (1593)	0.872
Alpha blockers	0.0% (1)	0.0% (0)	0.0% (1)	0.939
ARBs	16.6% (2558)	13.9% (1154)	19.7% (1404)	<0.001
Beta blockers	29.2% (4497)	25.7% (2126)	33.3% (2371)	<0.001
Calcium channel blockers	24.9% (3834)	21.4% (1774)	28.9% (2060)	<0.001
Digoxin	0.2% (36)	0.3% (26)	0.1% (10)	0.040
Diuretics	24.1% (3720)	20.7% (1719)	28.1% (2001)	<0.001
Heparin group	0.6% (98)	0.5% (39)	0.8% (59)	0.007
Nitrates	2.9% (447)	3.2% (267)	2.5% (180)	0.012
Antiplatelets other than Aspirin	6.0% (927)	7.4% (617)	4.4% (310)	<0.001
Vasodilators	0.0% (4)	0.0% (2)	0.0% (2)	1

**CHF**: congestive heart failure; **IHD**: ischemic heart disease; **COPD**: chronic obstructive pulmonary disease; **AF**: atrial fibrillation; **MI**: myocardial infarction; **TIA**: transient ischemic attack; **ACE**: angiotensin converting enzyme; **ARB**: angiotensin receptor blockers, **SES**: socioeconomic status.

• Residential area socioeconomic score range between1 (lowest) to 10. See text for details.

**Fig 1:**
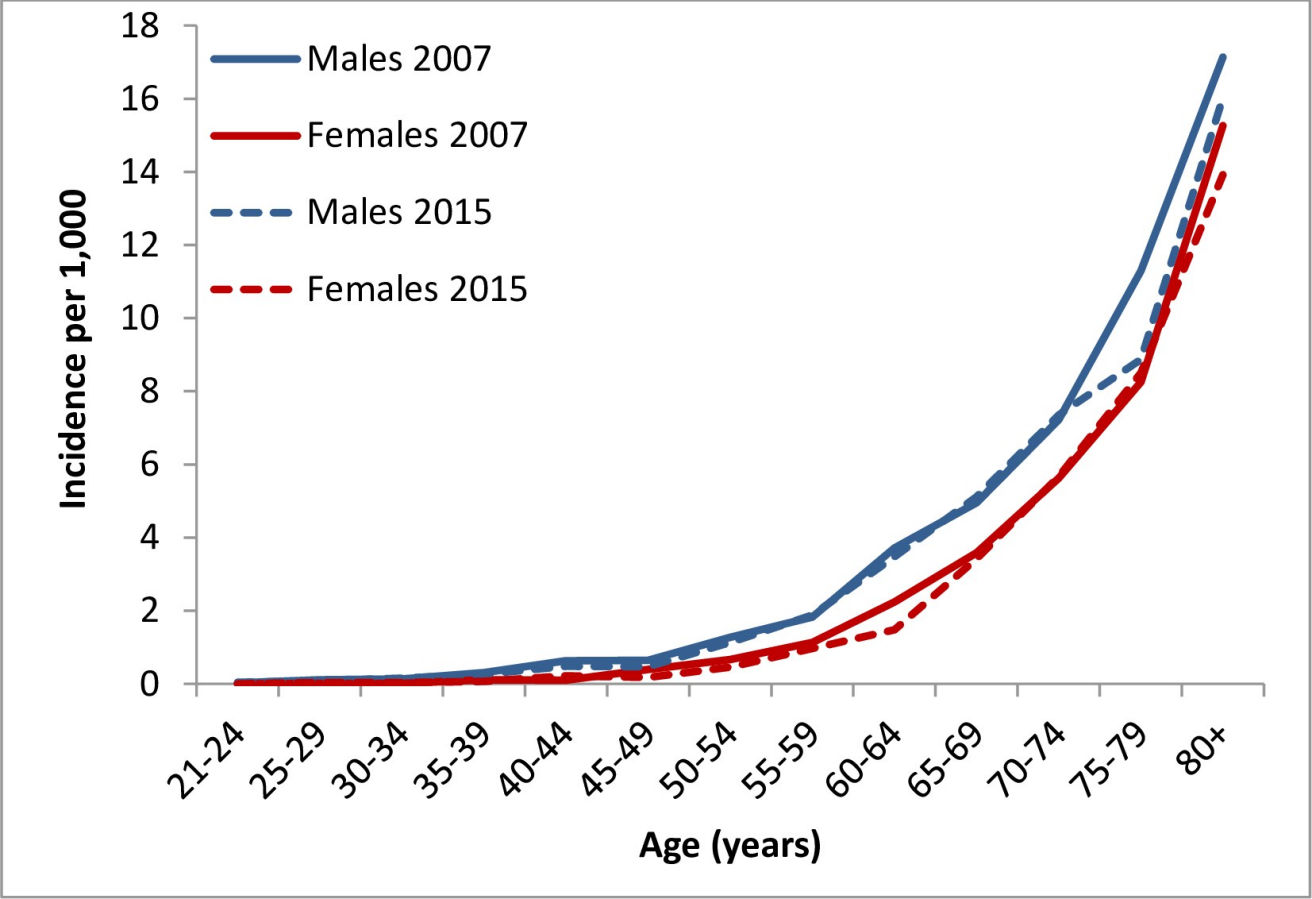
Incidence of AF by age and sex in 2007 and 2015.

**Fig 2:**
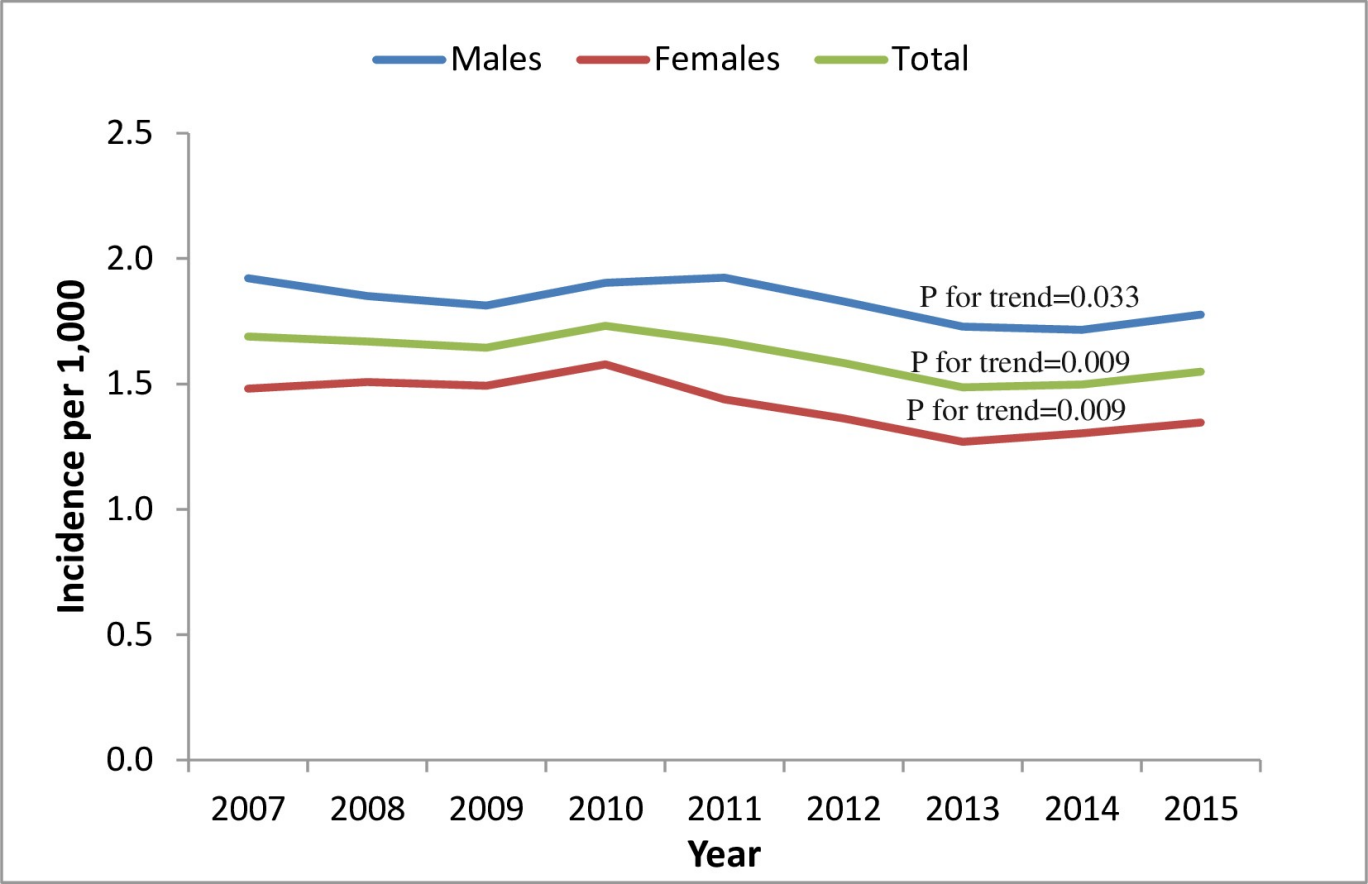
Temporal trends in age-adjusted incidence* of AF. *age-adjusted incidence evaluates the incidence that would have existed if the particular group under study had the same age distribution as the reference group. Thus, this measure adjusts for differences in incidence stemming for differences in age distributions.

The incidence of AF increased with age. Moreover, the incidence seemed to be higher in males compared with females across all age groups (Fig 1, S2 Table). Furthermore, the crude incidence increased over time from 1.35/1,000 person-years (PY) to 1.55/1,000 PY (males 1.53/1000 PY to 1.78/1000 PY, females 1.18/1000 PY to 1.35/1000 PY). The age-adjusted temporal trends in incidence are presented in Fig 2. A statistically significant decrease in the age-adjusted incidence was observed over time (-0.025/ 1000 PY, p = 0.009), both among males (-0.020/1000 PY, p-for trend = 0.033) and females (-0.025/1000 PY, p = 0.009).

### AF prevalence

**Fig 3:**
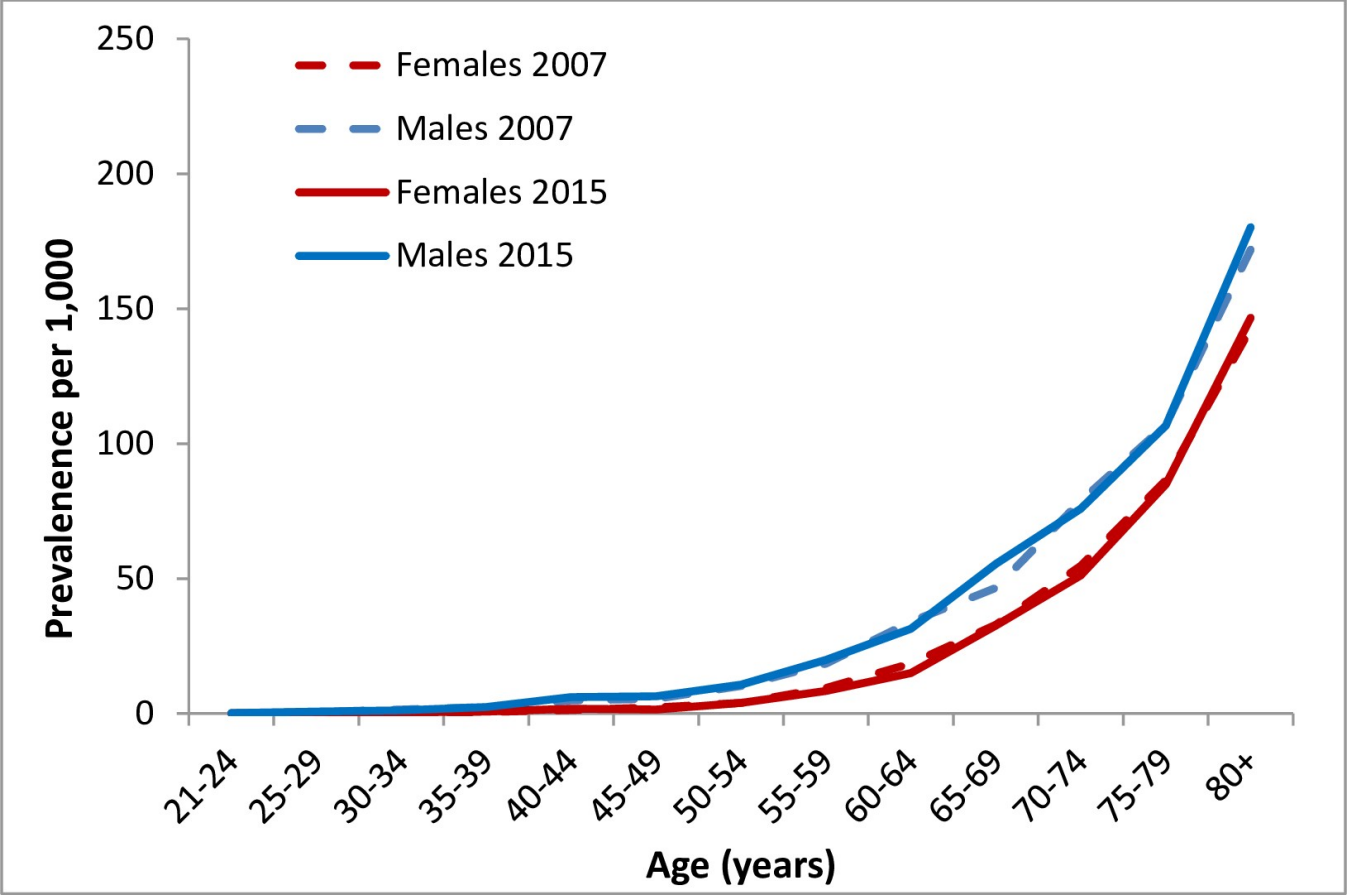
Prevalence of AF by age and sex in 2007 and 2015. * Every curve comprise a sample color halo represents 95% confidence interval.

**Fig 4:**
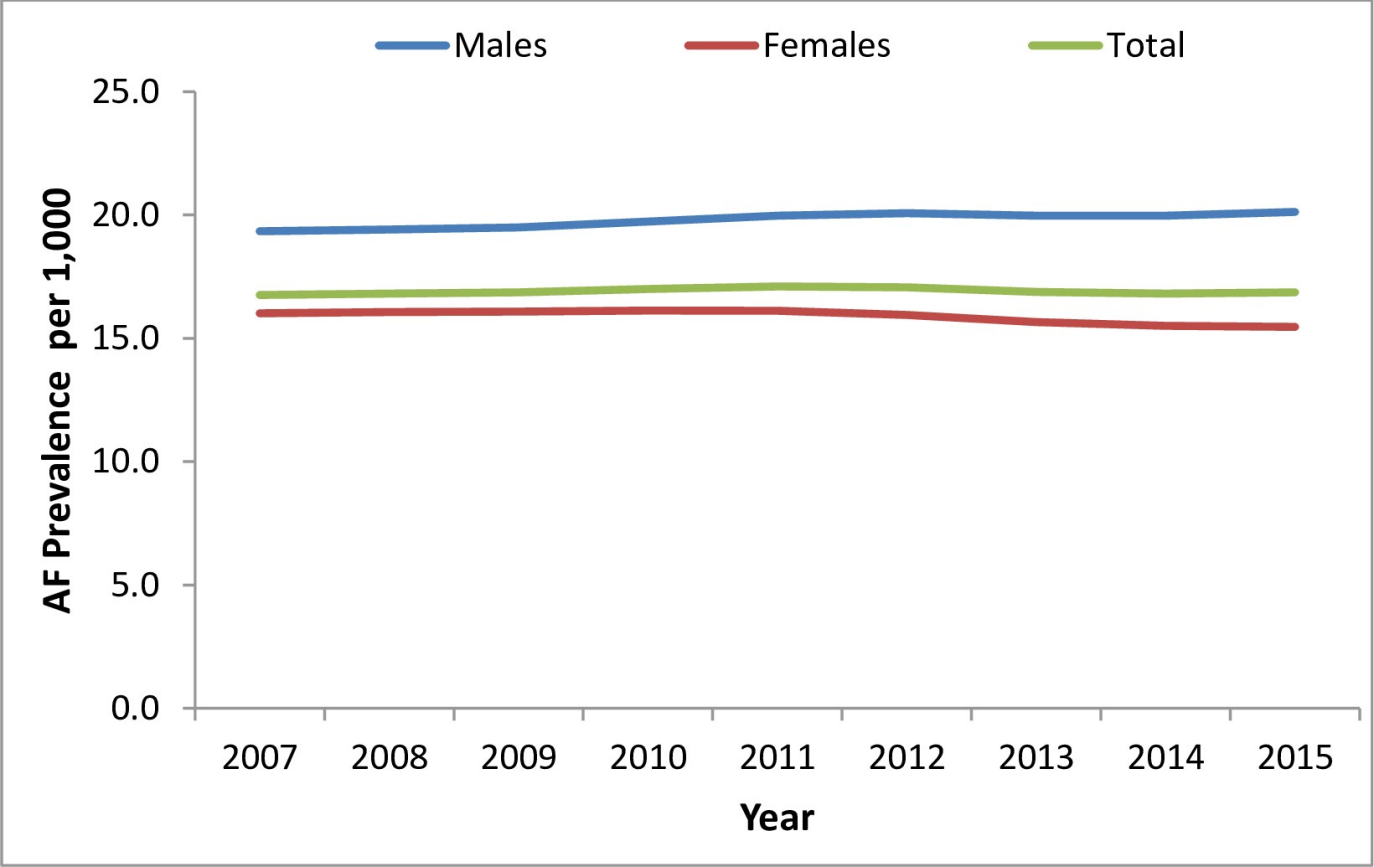
Temporal trends in age-adjusted prevalence of AF.

The total number of subjects diagnosed with AF increased from 14,527 (7,914 males, 6,613 females) in 2007 to 22,373 in 2015 (12,652 males, 9,721 females). This increase represents a total increment of 54%, with a greater increase in males (60%) than in females (47%). Fig 3 and S3 and S4 Tables present the number of subjects with AF stratified by sex and follow-up year and the prevalence of AF by age and sex, respectively. The prevalence of AF increased from 13.3/1,000 PY to 16.9/1,000 PY between 2007–2015. The prevalence of AF increased with age and seemed to be higher in males compared with females and higher in 2015 vs. 2007 for every age sub-group. A greater increase in the prevalence of AF was observed among males (15.3 to 20.1/1,000 PY) than among females (11.4 to 13.9/1,000 PY) over time. The age-adjusted temporal trends in AF prevalence presented in Fig 4 show no statistically significant difference over time (+0.010/1000 PY p = 0.554). However, a separate analysis of trends stratified by sex showed an increase in prevalence among males (+0.102/1000 PY, p-for trend<0.001) and a decrease among females (-0.082/1000 PY, p-for trend = 0.005).

### All-cause mortality in AF patients

**Fig 5:**
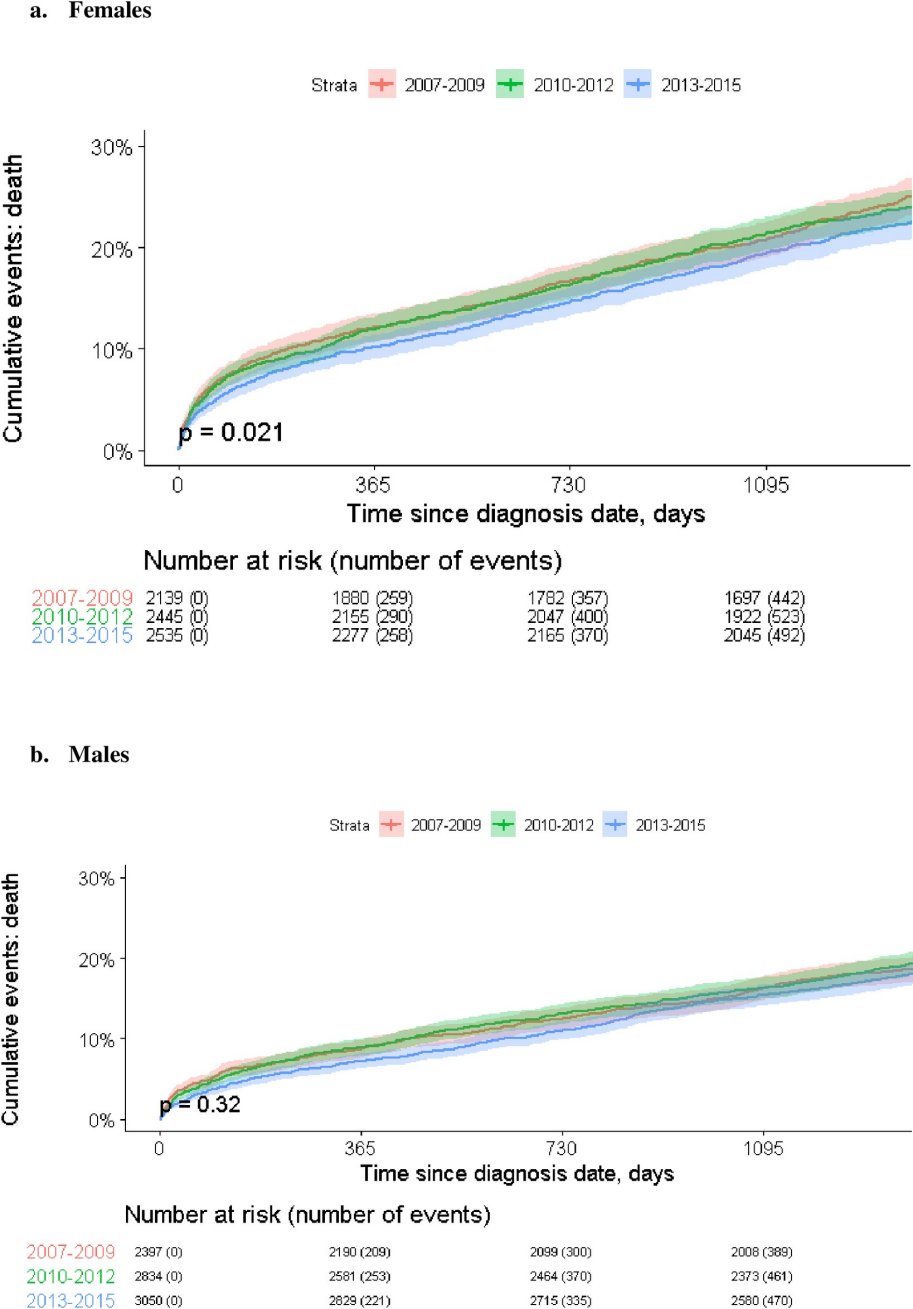
Mortality by AF diagnosis period in a. females and b. males. * Every curve comprises a sample color halo which represents 95% confidence interval.

Long term survival curves following AF diagnosis by sex are presented in S1 Fig. The five-year survival rate with AF was significantly higher among males compared to females (77.1% vs. 71.5%, respectively, p<0.001). Fig 5 presents sex-specific long-term survival curves, stratified by the period of diagnosis. In a multivariable Cox proportional hazards regression, male sex was statistically significantly (p = 0.002) associated with a HR of 0.89 (95%CI: 0.82–0.96) after adjusting for age, calendar year at index date, IHD, MI, stroke, HF, DM, CKD, hypertension, and osteoporosis.

As displayed in Fig 5, a statistically significant trend of improvement in survival was found among females but not males, throughout the study period.

## Discussion

The present study evaluated temporal trends in the incidence and prevalence of AF by age and sex in contemporary real-world population data obtained from a large healthcare provider in Israel. The main findings include the following: 1) the incidence of AF is higher in men versus women and increased over time in both sexes; however, the age-adjusted incidence decreased in both sexes. 2) Significant differences in the characteristics of the incident cases between sexes. Male patients were younger, had higher SES, higher prevalence of cardiovascular risk factors and comorbidities, yet lower CHA_2_DS_2_-VASC scores. This could be attributed to the higher scoring of female sex and older age among females rather than other comorbidities and risk factors in women. 3) The prevalence of AF increased with age, was higher in males compared with females, and seems to have increased in every age sub-group between 2007–2015. The latter increase was more pronounced among males compared with females. However, the age-adjusted analysis showed an increase in prevalence among males and a decrease among females over time. 4) Although overall long-term survival following AF diagnosis was better among men compared with women, a statistically significant trend for improved survival over time was found only in women.

### Incidence

Our findings are consistent with most other reports demonstrating a higher incidence of AF (up to 2-fold) among men compared with women and a disproportionate increase in incidence with increasing age among both sexes [12, 13, 17, 24, 25]. The incidence rates and the observed sex-related disparities in the characteristics of incident AF patients, particularly the older age, greater prevalence of hypertension, and the lower prevalence of IHD among females, are overall consistent with rates reported from other developed countries [17, 26–28].

Several previous studies have evaluated temporal trends in the sex-specific incidence and AF, most showing findings consistent with ours [12, 13, 18, 29]. The Framingham Heart Study and a global systematic review reported an increase in the incidence of AF in both sexes between 1958–2007 and 1990–2010, respectively. [13, 17]. However, recent studies have suggested plateauing and flattening of the trend of an increase in the incidence of AF [29–32], which are in partial agreement and possibly precede our findings of the age-adjusted decrease in the incidence of AF. Our observation is novel and significant, for which a clear explanation is unknown. Possible explanations could be related to “technical issues” such as the contemporary period of our study (advanced diagnostic methods available throughout the entire period), the age-selective intensity of diagnostic evaluation and documentation (greater in older people), reduced detection and recording of more sporadic forms of AF and methodological variations between studies. Alternatively, the age-adjusted decrease could potentially stem from an actual improvement in the “general health” of the population with better control of risk factors and AF shifting towards older age [33]. Moreover, it has been previously reported that the population attributable risk for AF of coronary disease is higher in men, whereas the population attributable risks of elevated systolic blood pressure and valvular disease are higher in women [19, 34]. Hence, different trends in the incidence and management of such risk factors could result in sex-related disparities in the incidence of AF.

### Prevalence

Similar to incidence, the prevalence of AF was significantly greater in men compared with women. This finding is consistent with previous reports from other countries, although AF prevalence has been shown to differ significantly according to ancestry [17, 18]. Contemporary studies evaluating sex-specific temporal trends in AF prevalence are relatively scarce. Two studies from the UK, based on primary care settings, showed an increase in AF prevalence in both sexes across all ages. [35, 36]. Similar findings also reported in a 50-year follow-up from the Framingham cohort and a community-based study from Iceland [13, 18]. Our age-adjusted prevalence trends showed an increase among men and a decline in women. Such sex-related disparities are in agreement with previous findings from the Copenhagen City Heart Study [37]. Hence, our results, which are based on a relatively contemporary cohort, could be one of the first indications supporting the notion that prevalence is indeed leveling off. Two main determinants of AF prevalence, which can explain the observed trends, are the incidence of AF and improved survival. With the incidence plateauing, the increased survival and aging of the patients could be more prominent explanations for the elevated unadjusted prevalence. Moreover, earlier diagnosis (i.e., lead-time bias) resulting from increased awareness and more intensive and efficient monitoring could also explain, in part, increasing prevalence.

Numerous sex-specific factors could account for the differences in the incidence, prevalence, and temporal trends of AF between men and women. First, sex differences in risk factors, comorbidities, higher frequency of atypical presentation, and deferred diagnosis among women versus men [13, 26, 38]. Second, hormonal (increased estrogen, cyclical progesterone, and increased prevalence of thyroid abnormalities in women) and differences in cardiac electrophysiological parameters (e.g. shorter atrial myocyte action potential duration, higher negative resting membrane potential, differences in K+ and Ca2+ currents among females) may all impact the risk for AF and sex-related differences [26, 39–41]. Third, structural differences between men and women. For example, smaller left atria and ventricles, lower left ventricular wall thickness, and a higher degree of atrial fibrosis in women vs. men [42–44]. Fourth, metabolic differences could also play a role, with women reported having higher serum values of FGF-23 and inflammatory markers such as C-reactive protein level [26, 43, 45]. Fifth, sex-related differences in AF management, with a lower rate of pharmacological (antiarrhythmic and anticoagulants medications) or interventional rhythm control (electric cardioversion, or catheter ablation) among women compared with men [26, 28, 46]. Lastly, increased rate of complications such as stroke and thromboembolism and mortality among women [26, 46].

The explanations for the differences in temporal trends are not completely clear and are probably multifactorial, relating to differences in survival, risk factors, management, and diagnosis.

### Mortality

We found higher mortality rates among women compared with men; however, survival improved significantly only among women throughout the study period. The finding of higher mortality rates among women is consistent with a previous report from the Framingham Heart Study [47] and a large meta-analysis that included over 4 million patients [48]. Possible explanations of this finding include increased comorbidity, delayed AF diagnosis with a longer AF history, and a higher AF burden among women. Another plausible explanation is undertreatment, with multiple reports indicating a reduced rate of oral anticoagulants prescription, increased utilization of rate control over rhythm control strategy, and lower referral to invasive strategies (e.g., catheter ablation) alongside with increased rate of complications in these procedures [28, 49, 50].

Previous studies have demonstrated a trend for improved survival in AF over time, as shown in our investigation among females [13, 25, 29]. The upward trend in survival can be due to advances in medical and interventional management, particularly, the increasing use of safer and more effective oral anticoagulation (associated with reduction in the incidence of stroke and systemic embolism) [13, 32], earlier detection [32], and a particular focus on the management monitoring of women following the growing knowledge of their worse outcomes. Nevertheless, since women presenting with AF significantly differ from men, being older, with a higher prevalence of hypertension, heart failure with preserved ejection fraction, AF-related symptoms, and lower prevalence of CAD. It is possible, therefore, that growing emphasis on better guideline-directed management of cardiovascular risk factors, hypertension in particular, and not necessarily the AF-specific management, resulted in improved survival in women only [33, 51].

Several limitations should be acknowledged when interpreting our results. The present study is retrospective and observational and is subject to limitations inherent in such a design. Patient data were derived from one HMO in Israel, and although it is the second-largest HMO, selection bias is a likelihood, limiting thereby generalizability of findings. Since the diagnoses were primarily abstracted from administrative databases (based on ICD-9), inaccuracies from coding errors could potentially bias the results. Although the current study did not include adjudication and overruling of the diagnoses, such quality assurance measures are continuously utilized by MHS. Finally, asymptomatic AF or AF in patients who did not seek medical care was not recorded and accounted for, potentially underestimating AF incidence and prevalence. However, this drawback is common in most large-scale registries evaluating temporal trends in AF epidemiology.

## Conclusions

The incidence and prevalence of AF in Israel increased with age and were higher in men compared with women. Between 2007 and 2015, an increase in AF incidence and prevalence was observed in both but was more pronounced among men. However, after age-adjustment, the incidence of AF decreased in both sexes, while AF prevalence increased among males and decreased among females over time. Mortality rates were higher among women compared with men; however, survival improved significantly only among women throughout the study period. Further investigation of the observed trends and their causes as well as age and sex-sensitive programs targeted at optimizing evaluation and management of AF to reduce its burden and dire outcomes is warranted.

## Supporting information

S1 Fig Survival curves of patients diagnosed with AF by sex. (DOCX)

S1 Table Number of incident AF cases by year. (DOCX)

S2 Table AF incidence by age and sex in 2007 and 2015. (DOCX)

S3 Table Number of prevalent cases of AF by year. (DOCX)

S4 Table AF prevalence by age and sex in 2007 and 2015. (DOCX)
