# Supplementary figures and images for: Sex-specific contemporary trends in incidence, prevalence and survival of patients with non-valvular atrial fibrillation: A long-term real-world data analysis
Source: PLoS One. 2021 Feb 18;16(2):e0247097. doi: 10.1371/journal.pone.0247097 (PMC7891766; doi:10.1371/journal.pone.0247097)

**S1 Fig. Survival curves of patients diagnosed with AF by sex.**


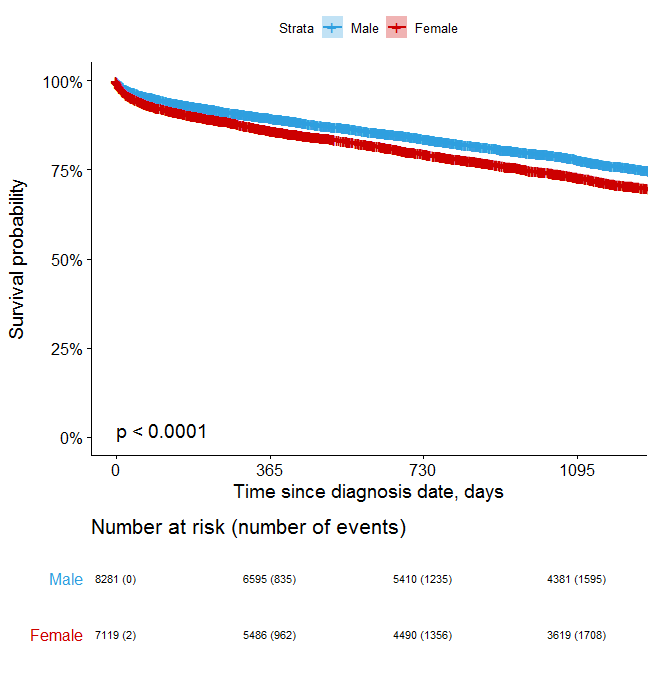

Supplement: S1 Fig — (DOCX) [file pone.0247097.s001.docx]
