# Supplementary material for: Sex-specific contemporary trends in incidence, prevalence and survival of patients with non-valvular atrial fibrillation: A long-term real-world data analysis
Source: PLoS One. 2021 Feb 18;16(2):e0247097. doi: 10.1371/journal.pone.0247097 (PMC7891766; doi:10.1371/journal.pone.0247097)
Supplement: S1 Table — (DOCX) [file pone.0247097.s002.docx]

S1 Table. Number of incident AF cases by year.

| **year** | **Males** | **Females** | **Total** |
| --- | --- | --- | --- |
| **2007** | 778 | 675 | 1,453 |
| **2008** | 795 | 713 | 1,508 |
| **2009** | 827 | 751 | 1,578 |
| **2010** | 915 | 851 | 1,766 |
| **2011** | 963 | 797 | 1,760 |
| **2012** | 958 | 797 | 1,755 |
| **2013** | 960 | 773 | 1,733 |
| **2014** | 1001 | 847 | 1,848 |
| **2015** | 1091 | 917 | 2,008 |
| **Total** | 8288 | 7121 | 15,409 |
