# Supplementary material for: Sex-specific contemporary trends in incidence, prevalence and survival of patients with non-valvular atrial fibrillation: A long-term real-world data analysis
Source: PLoS One. 2021 Feb 18;16(2):e0247097. doi: 10.1371/journal.pone.0247097 (PMC7891766; doi:10.1371/journal.pone.0247097)
Supplement: S2 Table — (DOCX) [file pone.0247097.s003.docx]

S2 Table. AF incidence by age and sex in 2007 and 2015.

| Age group | **2007** | | | | **2015** | | | |
| --- | --- | --- | --- | --- | --- | --- | --- | --- |
|  | **Males** | | **Females** | | **Males** | | **Females** | |
|  | # of cases | Incidence per 1,000 | # of cases | Incidence per 1,000 | # of cases | Incidence per 1,000 | # of cases | Incidence per 1,000 |
| **21-24** | 1 | 0.03 | 0 | 0.00 | 2 | 0.04 | 1 | 0.02 |
| **25-29** | 6 | 0.11 | 1 | 0.02 | 5 | 0.08 | 2 | 0.03 |
| **30-34** | 9 | 0.13 | 1 | 0.01 | 10 | 0.16 | 2 | 0.03 |
| **35-39** | 23 | 0.31 | 9 | 0.11 | 18 | 0.28 | 5 | 0.07 |
| **40-44** | 38 | 0.63 | 6 | 0.10 | 36 | 0.47 | 19 | 0.21 |
| **45-49** | 33 | 0.64 | 21 | 0.40 | 32 | 0.46 | 14 | 0.19 |
| **50-54** | 56 | 1.27 | 31 | 0.66 | 68 | 1.17 | 28 | 0.48 |
| **55-59** | 72 | 1.82 | 50 | 1.13 | 91 | 1.88 | 50 | 0.97 |
| **60-64** | 97 | 3.72 | 65 | 2.24 | 147 | 3.47 | 70 | 1.47 |
| **65-69** | 96 | 4.95 | 81 | 3.58 | 185 | 5.14 | 146 | 3.42 |
| **70-74** | 101 | 7.22 | 94 | 5.63 | 135 | 7.35 | 125 | 5.81 |
| **75-79** | 102 | 11.29 | 96 | 8.25 | 142 | 8.87 | 172 | 8.56 |
| **80-84** | 70 | 13.70 | 125 | 14.21 | 123 | 15.29 | 120 | 11.26 |
| **85-89** | 45 | 18.92 | 58 | 15.57 | 71 | 17.38 | 117 | 17.63 |
| **90-94** | 19 | 28.66 | 26 | 17.77 | 25 | 17.76 | 44 | 15.29 |
| **95+** | 10 | 39.68 | 11 | 25.76 | 6 | 15.83 | 13 | 19.64 |
| **Total** | 778 | 1.53 | 675 | 1.18 | 1096 | 1.78 | 928 | 1.35 |
| **Total N** | 1,459 | | | | 20,24 | | | |
| **Total 80+** | 144 | 17.14 | 220 | 15.26 | 225 | 16.17 | 294 | 14.11 |
| **Total Incidence** | 1.34 | | | | 1.55 | | | |
