# Supplementary material for: Sex-specific contemporary trends in incidence, prevalence and survival of patients with non-valvular atrial fibrillation: A long-term real-world data analysis
Source: PLoS One. 2021 Feb 18;16(2):e0247097. doi: 10.1371/journal.pone.0247097 (PMC7891766; doi:10.1371/journal.pone.0247097)
Supplement: S3 Table — (DOCX) [file pone.0247097.s004.docx]

S3 Table. Number of prevalent cases of AF by year.

| **year** | **Males** | **Females** | **Total** |
| --- | --- | --- | --- |
| **2007** | 7,914 | 6,613 | 14,527 |
| **2008** | 8,395 | 6,993 | 15,388 |
| **2009** | 8,935 | 7,400 | 16,335 |
| **2010** | 9,565 | 7,860 | 17,425 |
| **2011** | 10,181 | 8,250 | 18,431 |
| **2012** | 10,746 | 8,583 | 19,329 |
| **2013** | 11,302 | 8,865 | 20,167 |
| **2014** | 11,907 | 9,256 | 21,163 |
| **2015** | 12,652 | 9,721 | 22,373 |
