# Supplementary material for: Sex-specific contemporary trends in incidence, prevalence and survival of patients with non-valvular atrial fibrillation: A long-term real-world data analysis
Source: PLoS One. 2021 Feb 18;16(2):e0247097. doi: 10.1371/journal.pone.0247097 (PMC7891766; doi:10.1371/journal.pone.0247097)
Supplement: S4 Table — (DOCX) [file pone.0247097.s005.docx]

S4 Table. AF prevalence by age and sex in 2007 and 2015.

| Age group | **2007** | | | | **2015** | | | |
| --- | --- | --- | --- | --- | --- | --- | --- | --- |
|  | **Males** | | **Females** | | **Males** | | **Females** | |
|  | # of cases | Prevalence per 1,000 | # of cases | Prevalence per 1,000 | # of cases | Prevalence per 1,000 | # of cases | Prevalence per 1,000 |
| **21-24** | 6 | 0.15 | 0 | 0.00 | 7 | 0.15 | 2 | 0.04 |
| **25-29** | 26 | 0.48 | 12 | 0.19 | 44 | 0.68 | 18 | 0.27 |
| **30-34** | 104 | 1.48 | 28 | 0.33 | 75 | 1.20 | 27 | 0.40 |
| **35-39** | 182 | 2.45 | 48 | 0.60 | 152 | 2.35 | 55 | 0.73 |
| **40-44** | 295 | 4.87 | 85 | 1.38 | 464 | 5.99 | 155 | 1.75 |
| **45-49** | 284 | 5.48 | 116 | 2.19 | 447 | 6.44 | 112 | 1.54 |
| **50-54** | 451 | 10.14 | 184 | 3.91 | 623 | 10.59 | 233 | 3.95 |
| **55-59** | 742 | 18.50 | 419 | 9.40 | 975 | 19.80 | 435 | 8.36 |
| **60-64** | 914 | 33.98 | 544 | 18.45 | 1364 | 31.33 | 717 | 14.89 |
| **65-69** | 940 | 46.53 | 768 | 32.98 | 2102 | 55.44 | 1436 | 32.65 |
| **70-74** | 1187 | 78.73 | 959 | 54.63 | 1497 | 75.88 | 1152 | 51.10 |
| **75-79** | 1071 | 107.07 | 1092 | 86.47 | 1893 | 106.61 | 1850 | 84.95 |
| **80-84** | 907 | 152.82 | 1236 | 124.75 | 1495 | 158.74 | 1503 | 124.80 |
| **85-89** | 529 | 184.77 | 735 | 167.12 | 953 | 191.90 | 1214 | 156.99 |
| **90-94** | 202 | 238.77 | 282 | 164.53 | 445 | 243.44 | 655 | 187.79 |
| **95+** | 74 | 234.18 | 105 | 203.07 | 116 | 237.22 | 157 | 194.79 |
| **Total** | 7914 | 15.33 | 6613 | 11.44 | 12652 | 20.13 | 9721 | 13.93 |
| **Total N** | 14,527 | | | | 22,373 | | | |
| **Total 80+** | 1712 | 171.86 | 2358 | 142.62 | 3009 | 180.17 | 3529 | 146.61 |
| **Total pre-valence** | 13.27 | | | | 16.87 | | | |
